# Coenzyme-A-Independent Transacylation System; Possible Involvement of Phospholipase A2 in Transacylation

**DOI:** 10.3390/biology6020023

**Published:** 2017-03-30

**Authors:** Atsushi Yamashita, Yasuhiro Hayashi, Naoki Matsumoto, Yoko Nemoto-Sasaki, Takanori Koizumi, Yusuke Inagaki, Saori Oka, Takashi Tanikawa, Takayuki Sugiura

**Affiliations:** Faculty of Pharma-Sciences, Teikyo University, 2-11-1 Kaga, Itabashi-Ku, Tokyo 173-8605, Japan; hayashiy@pharm.teikyo-u.ac.jp (Y.H.); n-matsu@pharm.teikyo-u.ac.jp (N.M.); ynsasaki@pharm.teikyo-u.ac.jp (Y.N.-S.); zuml936@gmail.com (T.K.); onkoutokuzitu05790110@gmail.com (Y.I.); s.oka@pharm.teikyo-u.ac.jp (S.O.); tanikawa@pharm.teikyo-u.ac.jp (T.T.); sugiurat@pharm.teikyo-u.ac.jp (T.S.)

**Keywords:** CoA-independent transacylation system, acyltransferase, phospholipase A2, lysophospholipid, lysophospholipase/transacylation, platelet-activating factor, alkyl-ether linked phospholipid, eicosanoid, cPLA2γ

## Abstract

The coenzyme A (CoA)-independent transacylation system catalyzes fatty acid transfer from phospholipids to lysophospholipids in the absence of cofactors such as CoA. It prefers to use C20 and C22 polyunsaturated fatty acids such as arachidonic acid, which are esterified in the glycerophospholipid at the *sn*-2 position. This system can also acylate alkyl ether-linked lysophospholipids, is involved in the enrichment of arachidonic acid in alkyl ether-linked glycerophospholipids, and is critical for the metabolism of eicosanoids and platelet-activating factor. Despite their importance, the enzymes responsible for these reactions have yet to be identified. In this review, we describe the features of the Ca^2+^-independent, membrane-bound CoA-independent transacylation system and its selectivity for arachidonic acid. We also speculate on the involvement of phospholipase A2 in the CoA-independent transacylation reaction.

## 1. Introduction: Diversity of Fatty Acids in Glycerophospholipids

**Figure 1:**
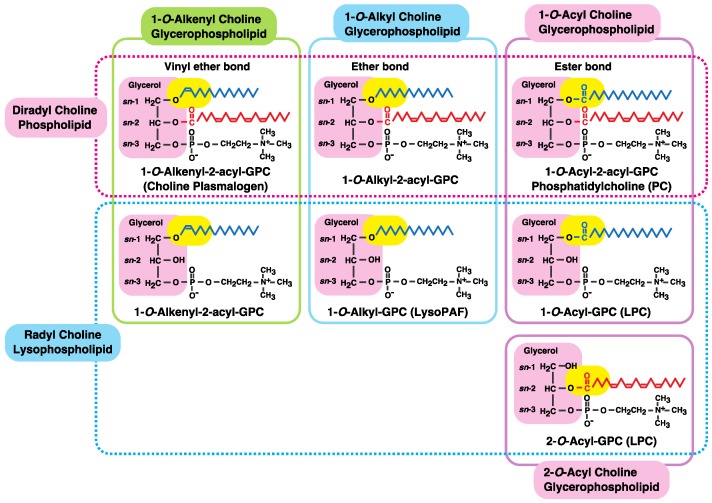
Diradyl choline glycerophospholipids and their lysophospholipids. The radyl moiety corresponds to an acyl, alkyl, or alkenyl group. All choline glycerophospholipids contain a glycerol backbone, which is shown on a pink background. The chemical linkage of the fatty chain at the *sn*-1 position of the backbone is shown on a yellow background. Each class of choline glycerophospholipid is further sub-classified into 1,2-diacyl, 1-*O*-alkyl-2-acyl, or 1-*O*-alkenyl-2-acyl types according to the chemical linkage of the fatty chain at the *sn*-1 position of the glycerol backbone. Their lysophospholipids are formed by fatty acid deacylation at the *sn*-2 position of the backbone. In the case of diradyl phospholipids, deacylation occurs at the *sn*-1 or -2 position, yielding a 2- or 1-acyl lysophospholipid, respectively. Fatty chains at the *sn*-1 and -2 positions are shown in blue and red, respectively.

Various types of glycerophospholipid classes with different polar head groups, such as choline, ethanolamine, serine, and inositol glycerophospholipids, are present in mammalian cells and tissues (Table 1). Each phospholipid is sub-classified into the diacyl, alkylacyl, or alkenylacyl type based on the chemical linkage of the fatty chain—i.e., acyl ester, ether, and vinyl ether bonds—at the *sn*-1 position of glycerol [1,2,3,4,5,6,7,8,9,10]. Figure 1 shows the chemical structures of diradyl phospholipids and their lysophospholipids as examples of choline glycerophospholipids. The radyl group can be an acyl, alkyl, or alkenyl group; diradyl phospholipids therefore include diacyl, alkylacyl, and alkenylacyl phospholipids. Radyl lysophospholipids are the deacylated form of each diradylphospholipid. Each sub-classified phospholipid is further grouped into distinct molecular species depending on the chain length and degree of unsaturation at the *sn*-1 and -2 radyl residues. Different combinations of head groups, *sn*-1 linkages, and fatty chains can yield several hundred different molecular species of glycerophospholipids in mammalian tissues [3,4,5,6,7,8,9,10,11,12,13].

## 2. Fatty Acid Remodeling System of Glycerophospholipids

**Figure 2:**
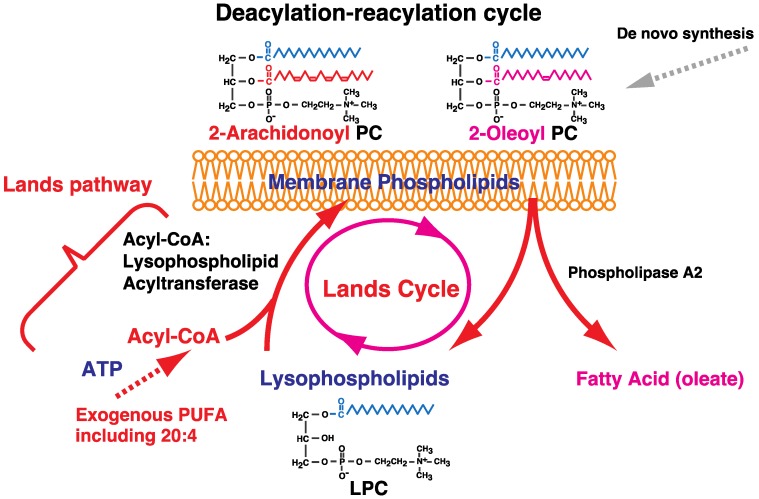
Fatty acid remodeling of phospholipids by the deacylation–reacylation cycle (Lands cycle). The Lands cycle begins with PLA2, which releases a fatty acid from a phospholipid, and then another fatty acid is incorporated into the phospholipid by an acyl-CoA:1-acyl lysophospholipid acyltransferase. Exogenous PUFAs, such as arachidonic acid, are incorporated into phospholipids during the cycle. Exogenous stearic acid is also concentrated at the *sn*-1 positions of phospholipids by a similar deacylation–reacylation cycle consisting of phospholipase A1 (PLA1) and acyl-CoA:2-acyl lysophospholipid acyltransferases. ATP is required for acyl-CoA synthesis in the reacylation step.

Various types of fatty acids are present in each glycerophospholipid at the *sn*-1 or -2 position of the glycerol backbone. Observation of differences in turnover rates of fatty acyl and glycerol moieties of phospholipids led to the identification of acyl-CoA:lysophospholipid acyltransferase in rat liver and the subsequent discovery of the fatty acid remodeling system (Table 2 and Figure 2) [14,15], which increases the diversity of fatty acids in phospholipids [1,2,3,4,5,6]. Fatty acids are first incorporated into glycerophospholipids during de novo synthesis; some polyunsaturated fatty acids (PUFAs) such as arachidonic acid (5,8,11,14-eicosatetraenoic acid, 20:4 n-6) are then incorporated by the remodeling system. Stearic acid (octadecanoic acid, 18:0) is also incorporated at the *sn*-1 position during the remodeling process.

The remodeling system includes acyltransferases and transacylation of lysophospholipids that catalyze fatty acid transfer between an acyl donor and acceptor lysophospholipid (Table 2) [4,5,6,7,8,9,10,11,12,13]. The acyl donors for acyl-CoA:lysophospholipid acyltransferases are fatty acyl-CoAs, the activated forms of fatty acids that facilitate fatty acid transfer to acceptor molecules. The activation of free fatty acids consumes ATP. In contrast, the transacylation system employs the esterified fatty acids in phospholipids as acyl donors that are transferred to acceptor lysophospholipids. This transacylation reaction is essentially ATP-independent.

Exogenous arachidonic acid is not typically introduced into phospholipids via de novo synthesis but is instead incorporated during fatty acid remodeling [3,4,5,6,7,8,9,10,11,12,13]. This is important for the biosynthesis of lipid messengers since arachidonic acid is the precursor of eicosanoids such as prostaglandins and leukotrienes. In de novo synthesis of phospholipids, fatty acids that are saturated or exhibit a low degree of unsaturation, such as oleic acid (9-octadecenoic acid, 18:1 n-9), are often incorporated at the *sn*-2 positions of phospholipids. Their hydrolysis by phospholipase A2 (PLA2) releases the fatty acid, and the resultant lysophospholipids are acylated by acyl-CoA:lysophospholipid acyltransferases. Since acyltransferases prefer PUFA-CoAs such as arachidonoyl-CoA, PUFAs accumulate in the *sn*-2 positions of phospholipids during the deacylation–reacylation cycle (Lands cycle) (Figure 2).

**Figure 3:**
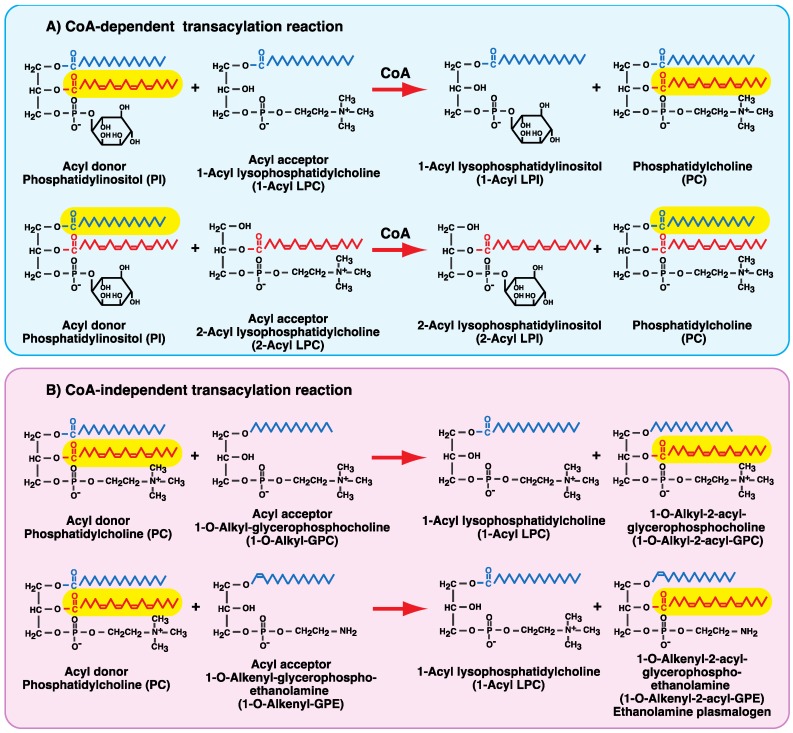
CoA-dependent and -independent transacylation reactions. (**A**) In CoA-dependent transacylation, *sn*-1 or -2 fatty acids are transferred between phospholipids and lysophospholipids in the presence of CoA. This reaction uses choline, ethanolamine, serine, and inositol glycerophospholipids as acyl donors and lysophospholipids as acyl acceptors. PI is a typical, highly effective acyl donor. (**B**) In CoA-independent transacylation, only *sn*-2 fatty acids are transferred, using choline and ethanolamine glycerophospholipids as acyl donors. This system preferentially employs 1-*O*-alkyl and 1-*O*-alkenyl choline and ethanolamine lysophospholipids as acceptors. 1-*O*-Alkyl-GPC and 1-*O*-alkenyl-GPE are represented as acyl acceptors because these lysophospholipids are effective acyl acceptors. The fatty chain at *sn*-1 and fatty acid at *sn*-2 are shown in blue and red, respectively; the transferred fatty acid is shown on a yellow background.

Two different transacylation systems (CoA-dependent and CoA/cofactor-independent) are also involved in fatty acid remodeling (Figure 3). CoA-dependent transacylation activity was first reported in rat liver microsomes [16]. In this reaction, esterified fatty acids in phospholipids are transferred to lysophospholipids to form phospholipids in the presence of CoA without generating free fatty acids [7,8,9,10,11,16,17,18,19,20,21,22,23]. CoA-dependent transacylation uses a variety of glycerophospholipids, such as phosphatidylcholine (PC) and phosphatidylinositol (PI), as acyl donors and lyso-type glycerophospholipids, such as lysophosphatidylcholine (LPC) and lysophosphatidylinositol (LPI), as acyl acceptors (Table 1). This activity is distributed in the membrane fractions of mammalian tissues and cells, including in microsomes. The K_m_ for CoA in the CoA-dependent transacylation reaction is very low (1–4 μM), suggesting that the reaction proceeds in the presence of physiological levels of CoA. We have proposed that the CoA-dependent transacylation reaction is mediated by a combination of reverse and forward reactions of acyl-CoA:lysophospholipid acyltransferases [7,8,23,24,25,26,27].

In contrast to CoA-dependent transacylation, the mechanisms of CoA-independent transacylation are not fully understood. In this review, we describe the properties and possible mechanisms of the CoA/cofactor-independent transacylation system. We hypothesize that this reaction is catalyzed by PLA2, as described in detail below.

## 3. Alkyl- or Alkenyl-Type Glycerophospholipids Contain Large Amounts of PUFAs, Such as Arachidonic Acid

As previously mentioned, choline and ethanolamine glycerophospholipids are sub-classified into diacyl, alkylacyl, and alkenylacyl types based on the chemical linkage of the fatty chains (i.e., acyl ester, ether, and vinyl ether bonds) at the *sn*-1 position of glycerol [3,5,6,7,8,9,10] (Figure 1 and Table 1). Serine and inositol glycerophospholipids in mammalian tissues are of the diacyl type.

**Figure 4:**
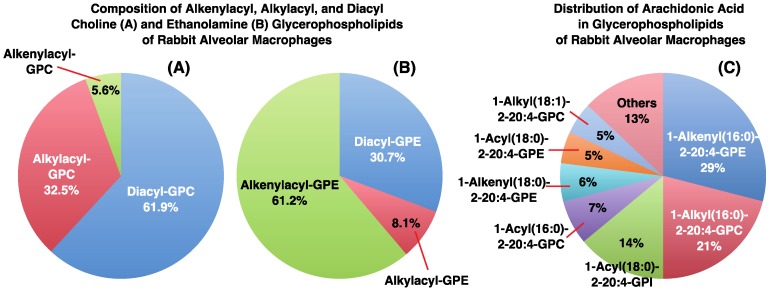
Composition of alkenylacyl, alkylacyl, and diacyl choline (**A**) and ethanolamine (**B**) glycerophospholipids and distribution of arachidonic acid in glycerophospholipids (**C**) of rabbit alveolar macrophages. Ether-linked phospholipids, including 1-*O*-alkyl-2-acyl and 1-*O*-alkenyl-2-acyl types, account for approximately 40% and 70% of choline and ethanolamine glycerophospholipids, respectively. Arachidonic acid (20:4) is mostly distributed in ether-linked phospholipids, including 1-*O*-alkyl-2-acyl-GPC and 1-*O*-alkenyl-2-acyl-GPE. Modified from Sugiura et al. [30].

Large amounts of ether-linked phospholipids, including 1-*O*-alkyl-2-acyl-glycerophosphocholine (1-*O*-alkyl-2-acyl-GPC) and 1-*O*-alkenyl-2-acyl- glycerophosphoethanolamine (1-*O*-alkenyl-2-acyl-GPE, ethanolamine plasmalogen), are present in various tissues (Table 1). In the human heart, nearly 30–40% of choline glycerophospholipids are 1-*O*-alkenyl-2-acyl-GPC (choline plasmalogen) [3,7,8]. Even more striking is the fact that almost 30% of the glycerophospholipids in the adult human brain and up to 70% of myelin sheath ethanolamine glycerophospholipids are ethanolamine plasmalogens [3,5,6,7,8,9,10,11,28]. In addition, high levels of 1-*O*-alkyl-2-acyl-GPC and ethanolamine plasmalogen are present in white blood cells such as polymorphonuclear leukocytes, macrophages, and eosinophils [3,5,6,7,8,9,10,11,28]. In rabbit alveolar macrophages, 1-*O*-alkylacyl-GPC and 1-*O*-alkenylacyl-GPE account for 32.5% and 61.2% of choline and ethanolamine glycerophospholipids, respectively (Figure 4A,B) [3,5,6,7,8,9,10,11,28].

Arachidonic acid (20:4n-6), a common precursor of eicosanoids, is usually esterified at the *sn*-2 position in each phospholipid class [3,5,6,7,8,9,10,11,28]. Notably, arachidonic acid levels are higher in ether-linked phospholipids than in their diacyl counterparts. In rabbit alveolar macrophages, a significant proportion of arachidonic acid is located at the *sn*-2 positions of 1-*O*-alkyl-2-acyl-GPC and 1-*O*-alkenyl-2-acyl-GPE (Figure 4C) [28]. In human eosinophils, large amounts of arachidonic acid are esterified to 1-*O*-alkyl-2-acyl-GPC (92.0% in choline glycerophospholipids) and 1-*O*-alkenyl-2-acyl-GPE (86.6% in ethanolamine glycerophospholipids) [29].

The functions of ether-linked phospholipids have not yet been fully elucidated. The alkyl ether bond is more chemically stable than the ester bond and resistant to hydrolysis by esterases, including phospholipases. The vinyl ether bond (alkenyl ether bond) is susceptible to acid but resistant to phospholipases. The lack of the carbonyl oxygen at the *sn*-1 position affects the hydrophilicity of the headgroup and allows stronger intermolecular hydrogen bonding between the headgroups of the lipid. These properties favor the formation of non-lamellar structures that are expressed in the high-affinity structure of ethanolamine plasmalogen, allowing it to adopt an inverse hexagonal form [31].

Plasmalogens are present in various human tissues of the nervous and cardiovascular systems and are especially enriched in the myelin sheath, where they may contribute to membrane insulation. The vinyl ether bonds of plasmalogens can protect mammalian cells against the damaging effects of reactive oxygen species; reduced levels of brain tissue plasmalogens have been associated with Alzheimer’s disease [32], X-linked adrenoleukodystrophy [33], and defects in central nervous system myelination [34].

An important function of ether-linked membrane phospholipids is arachidonic acid storage. As mentioned earlier, arachidonic acid is highly enriched in ether-linked phospholipids such as 1-*O*-alkyl-2-acyl-GPC and 1-*O*-alkenyl-2-acyl-GPE (Figure 4C). The former is the precursor of platelet-activating factor (PAF), a potent bioactive mediator. The synergistic synthesis of PAF and eicosanoids is discussed in Section 6.

## 4. CoA-Independent Transacylation Activity between Diacyl Phospholipids and Ether-Linked Lysophospholipids

The CoA-independent transacylation reaction (Figure 3B) was first described in human platelets [35,36]. Similar transacylation activity was also detected in microsomes and the membrane fractions of several mammalian cells and tissues, including macrophages [17,18,19,37,38], neutrophils [39,40], and platelets [41] and the brain [42,43], heart [44], testis [45], and Ehrlich cells [46]. This reaction catalyzes fatty acid transfer from diacyl-type glycerophospholipids to a variety of lysophospholipids even in the absence of cofactors; this is in contrast to CoA-dependent transacylation, which requires CoA. CoA-independent transacylation does not require a divalent cation such as Ca^2+^. 1-Radyl-2-lyso-GPC and 1-radyl-2-lyso-GPE (Figure 3B) but not 1-acyl-glycerophosphoinositols (1-acyl-GPI and 1-acyl LPI), 1-acyl-glycerophosphoserines (1-acyl-GPS and 1-acyl lysophosphatidylserine), or 1-acyl-glycerophosphates (1-acyl-GP and 1-acyl lysophosphatidic acid) act as acyl acceptors; thus, the headgroups of the acceptor lipids are limited to phosphocholine and phosphoethanolamine [17,18,25]. For fatty chain linkage at the *sn*-1 position of the acyl acceptor glycerol, three subclasses—1-*O*-alkenyl-GPC, 1-*O*-alkyl-GPC, and 1-acyl-GPC—have been shown to be effective acceptors, with 1-*O*-alkyl-GPC undergoing the most rapid acylation. 1-*O*-Alkenyl-GPE, 1-*O*-alkyl-GPE, and 1-acyl-GPE are also acylated with arachidonic acid transferred from diacyl-GPC [18,25].

Regarding donor glycerophospholipids, choline glycerophospholipids—particularly diacyl-GPC—are the preferred substrate [17,18,25]. Although diacyl-GPE also serves as a donor, diacyl-GPI is not involved when 1-*O*-alkyl-GPC is the acceptor. In terms of the positional specificity of fatty acids in the glycerol backbone, fatty acids esterified at the *sn*-2 but not the *sn*-1 positions of diacyl glycerophospholipids participate in CoA-independent transacylation. In contrast, free fatty acids and acyl-CoAs are not used as acyl donors. Fatty acids transferred by the CoA-independent transacylation system are limited to C20/C22 chain-length PUFAs, such as 8,11,14-eicosatrienoic acid (20:3n-6, dihomo-γ-linolenic acid), arachidonic acid (20:4n-6), 5,8,11,14,17-eicosapentaenoic acid (20:5n-3), 4,7,10,13,16-docosapentaenoic acid (22:4n-6), and 4,7,10,13,16,19-docosahexaenoic acid (22:6n-3, DHA); both *n*-6 and *n*-3 fatty acids can be transferred [17,18,19,35,36,37,43].

The substrate specificity and tissue distribution of CoA-independent transacylation explain the complement of alkyl ether-linked phospholipids in mammalian cells and tissues. Large amounts of ether-linked phospholipids, including 1-*O*-alkyl-2-acyl-GPC and 1-*O*-alkenyl-2-acyl-GPE, are detected in tissues such as the brain and heart and in inflammatory cells such as neutrophils, macrophages, platelets, and lymphocytes. Although a high proportion of arachidonic acid (20:4 n-6) is found at the *sn*-2 positions of ether-linked glycerophospholipids [6], arachidonoyl-CoA acyltransferase activity for 1-*O*-alkyl-GPC and 1-*O*-alkenyl-GPC is very low [47,48,49]. In contrast, CoA-independent transacylation was observed in the membrane fractions of various mammalian tissues excluding the liver [17,18], in which ether-containing phospholipids are almost completely absent [3]. The tissue distribution of CoA-independent transacylation activity appears to be closely related to the amounts of ether-linked glycerophospholipids in these tissues. The fatty acid specificity of this activity in vitro reflects the fatty acid pattern at the *sn*-2 positions of ether-linked glycerophospholipids [3,11,18]. CoA-independent transacylation is assumed to play an important role in the acylation of ether-linked lysophospholipids to generate PUFA-containing ether-linked glycerophospholipids.

There are few studies on the upregulation of CoA-independent transacylation activity. Treatment of platelets with phorbol ester or diacylglycerol was shown to enhance CoA-independent transacylation [50], which was also increased by treatment of human neutrophils with tumor necrosis factor-α [51].

Two structurally distinct compounds, SK&F 98625 [diethyl 7-(3,4,5-triphenyl-2-oxo-2,3- dihydro-imidazole-1-yl)heptane phosphonate] and SK&F 45905 [2-[2-(3-4-chloro-3- (trifluoromethyl)phenyl)-ureido]-4-(trifluoromethyl)phenoxy]-4,5-dichlorobenzenesulfonic acid], have been shown to inhibit CoA-independent transacylation [52] via competition with acceptor lysophospholipids (IC_50_: 6–19 μM) while having little or no effect on related enzymes including lysoPAF acetyltransferase, 14-kDa secretory PLA2, and cyclooxygenase. The antiproliferative agent ET-18-O-CH3 (1-*O*-octadecyl-2-*O*-methyl-GPC) similarly inhibits CoA-independent transacylation (IC_50_, 0.5 μM) [53]. These compounds have been shown to inhibit proliferation and induce apoptosis in HL-60 monocytic leukemia cells, suggesting a link between CoA-independent transacylation, remodeling of ether-containing phospholipids with arachidonic acid, and leukemia cell survival. Because SK&F 98625 and SK&F 45905 exhibited potent inhibition of 5-lipooxygease and modest inhibition of acyl-CoA:lysophospholipid acyltransferase, respectively [52], further development of specific inhibitors is needed.

## 5. Incorporation and Mobilization of Arachidonic Acid in Lipid Subclasses via Acyl-CoA Acyltransferases and CoA-Independent Transacylation

**Figure 5:**
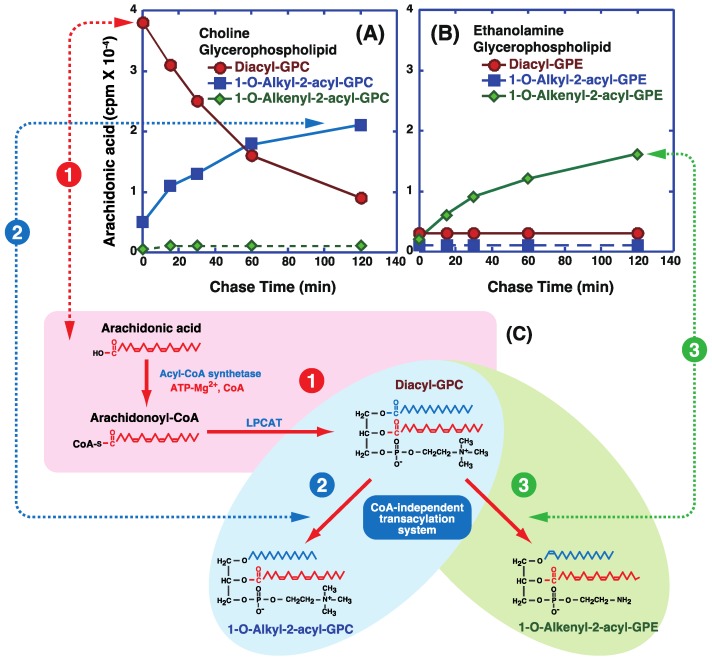
Incorporation and mobilization of arachidonic acid in lipid classes of rabbit alveolar macrophages. (**A**,**B**) Macrophages were pulse-labeled with radioactive arachidonic acid for 7.5 min. After removal of labeled fatty acids, cells were chased for the indicated periods. The radioactive arachidonic acid in 1-*O*-alkenyl-2-acyl (green diamonds), 1-*O*-alkyl-2-acyl (blue squares), and 1,2-diacyl (red circles) choline glycerophospholipids (**A**) and ethanolamine glycerophospholipids (**B**) was measured. (**C**) Schematic illustration of the incorporation and mobilization of arachidonic acid in different lipid classes. Arachidonic acid was first incorporated into diacyl-GPC via sequential reactions of acyl-CoA and acyl-CoA:1-acyl-GPC acyltransferase (one in red circle). Arachidonic acid in diacyl-GPC was then transferred to 1-*O*-alkyl-GPC (two in blue circle) and 1-*O*-alkenyl-GPE (three in green circle) by CoA-independent transacylation. Modified from Sugiura et al. [54].

The behavior of arachidonic acid in cells, including its incorporation into each lipid class, highlights the distinct roles and substrate specificities of acyl-CoA:lysophospholipid acyltransferases and the CoA-independent transacylation system. Exogenously added arachidonic acid is primarily incorporated into phospholipids in various cell types. Radiolabeled arachidonic acid was mainly incorporated into the diacyl-GPC subclass in intact rabbit alveolar macrophages (Figure 5A, red circles after a brief incubation (7.5 min) [54,55]. However, very little was incorporated into ether-linked phospholipids (Figure 5A,B, blue squares and green diamonds). Incorporation of arachidonic acid into diacyl-GPC during short-term incubation is thought to occur via sequential reactions of acyl-CoA synthetase and acyl-CoA:1-acyl-GPC acyltransferase (Lands pathway; Figure 2). The fact that ATP, CoA, and Mg^2+^ are required for the formation of acyl-CoA from free fatty acids by acyl-CoA synthetase indicates that energy is needed for fatty acid incorporation via the Lands pathway.

In pulse-chase-labeled macrophages, esterified arachidonic acid in the diacyl-GPC subclass was gradually transferred to 1-*O*-alkyl-2-acyl-GPC (Figure 2A, blue squares) and 1-*O*-alkenyl-2-acyl-GPE (Figure 2B, green diamonds) [54,55]. Similar incorporation and mobilization of arachidonic acid have been observed in neutrophils [39,40], platelets [41,56], HL-60 cells [57], and endothelial cells [58,59]. Esterified DHA (22:6n-3) in the diacyl-GPC subclass was also transferred to 1-*O*-alkyl-2-acyl-GPC and 1-*O*-alkenyl-2-acyl-GPE in alveolar macrophages [38].

However, although linoleic acid (9,12-octadecadienoic acid, 18:2n-6) was rapidly incorporated into subclasses of diacyl-GPC and diacyl-GPE via the Lands pathway, subsequent mobilization of linoleic acid from diacyl-GPC and diacyl-GPE subclasses to 1-*O*-alkyl-2-acyl-GPC and 1-*O*-alkenyl-2-acyl-GPE was not observed [54]. The transfer of arachidonic acid or DHA from diacyl-GPC to 1-*O*-alkyl-2-acyl-GPC and 1-*O*-alkenyl-2-acyl-GPE can be explained by CoA-independent activity, since the fatty acid and acceptor specificities resemble those of the CoA-independent transacylation reaction in in vitro cell-free assays. Such gradual transfers may account for the accumulation of arachidonic acid or DHA in ether-linked glycerophospholipids, such as 1-*O*-alkyl-2-acyl-GPC and 1-*O*-alkenyl-2-acyl-GPE, in inflammatory cells [18,54].

The incorporation of exogenous arachidonic acid into PI was also observed during short-term incubation [54,55]. However, the arachidonic acid in PI did not serve as an acyl donor in the CoA-independent transacylation system.

## 6. Involvement of the CoA-Independent Transacylation System in PAF Metabolism

**Figure 6:**
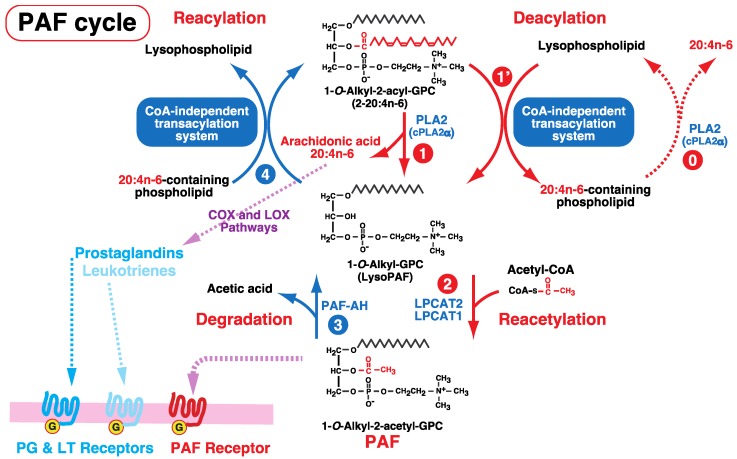
Biosynthesis and degradation of PAF. The PAF precursor 1-*O*-alkyl-2-acyl-GPC is hydrolyzed by a PLA2 protein such as cPLA2α to form 1-*O*-alkyl-GPC (lysoPAF) (Reaction 1). LysoPAF is also formed by the CoA-independent transacylation system through fatty acid transfer from 1-*O*-alkyl-2-acyl-GPC to a lysophospholipid (Reaction 1’). The lysophospholipid acceptor for the CoA-independent transacylation system is formed by hydrolysis mediated by a PLA2 protein such as cPLA2α (Reaction 0). The bioactive phospholipid PAF is formed by lysoPAF acetyltransferase (LPCAT1 and LPCAT2) through the reacetylation of lysoPAF. PAF activates the PAF receptor to trigger intracellular signaling. Because arachidonic acid is formed by PLA2 in the deacylation step (Reactions 1 and 0), eicosanoids, prostaglandins (PGs), and leukotrienes (LTs) are synthesized simultaneously by the COX and LOX pathways. PGs and LTs activate PG and LT receptors. PAF and PGs/LTs simultaneously activate the cells. In contrast, PAF is degraded through the deacetylation process by PAF acetylhydrolase (PAF-AH, Reaction 3). The resultant lysoPAF is further converted to 1-*O*-alkyl-2-acyl-GPC through the CoA-independent transacylation system (Reaction 4). The reactions involved in the synthesis of PAF are indicated in red, and those involved in the degradation of PAF are indicated in blue.

PAF was originally identified as a potent bioactive phospholipid mediator released from IgE-stimulated basophils that can induce aggregation of rabbit platelets [60]. Its chemical structure was identified as 1-*O*-alkyl-2-acetyl-*sn*-GPC [60,61,62,63]. PAF is a biologically active glycerophospholipid that is presumed to mediate inflammatory responses, including anaphylaxis and septic shock, through the seven transmembrane-type G protein-coupled PAF receptor (Figure 6) [64].

In inflammatory cells including neutrophils, eosinophils, and macrophages, PAF is mainly synthesized via the so-called remodeling (deacylation–reacetylation) pathway (Figure 6) [3,4,5,6,7,8,9,10,11]. Inflammatory cells contain large amounts of the PAF precursor 1-*O*-alkyl-2-acyl-GPC [3,5,7,8]. Arachidonic acid accumulates in the *sn*-2 position of 1-*O*-alkyl-2-acyl-GPC; 1-*O*-alkyl-2-arachidonoyl-GPC is hydrolyzed by cytosolic PLA2α (cPLA2α) to form 1-*O*-alkyl-GPC (lysoPAF) and arachidonic acid (Figure 6) [65,66,67]. Subsequently, lysoPAF acetyltransferases (LPCAT2 and LPCAT1) transfer acetate from acetyl-CoA to the *sn*-2 position of lysoPAF to generate PAF (Figure 6, reacetylation) [68,69,70].

CoA-independent transacylation also triggers PAF biosynthesis through the formation of lysoPAF (Figure 3, deacylation). The addition of 1-*O*-alkenyl-GPE (lysoplasmalogen) has been shown to increase the amount of PAF produced by opsonized zymosan-stimulated polymorphonuclear leukocytes, although 1-*O*-alkenyl-GPE is not a direct precursor of PAF synthesis [71]. The addition of 1-*O*-alkenyl-GPE to cells induces the formation of lysoPAF from 1-*O*-alkyl-2-arachidonoyl-GPC. Other lysophospholipids including LPC (1-acyl-GPC) and lysophosphatidylethanolamine (LPE, 1-acyl-GPE) also enhance lysoPAF production via CoA-independent transacylation (Figure 6, deacylation). Upon activation of cPLA2α by inflammatory stimuli, not only lysoPAF but also 1-*O*-alkenyl-GPE and other choline and ethanolamine lysophospholipids are formed, since all choline and ethanolamine glycerophospholipids serve as cPLA2α substrates. This increase in lysophospholipids leads to the generation of lysoPAF via CoA-independent transacylation followed by an increase in PAF generation [71,72,73].

PAF is degraded (deacetylated) by PAF acetylhydrolase (PAF-AH) (Figure 6, degradation) [74,75,76] to yield lysoPAF, which is further acylated by CoA-independent transacylation to regenerate the PAF precursor 1-*O*-alkyl-2-arachidonoyl-GPC (Figure 6, reacylation).

PAF and eicosanoids are simultaneously formed in inflammatory cells [3,4,5,6,7,8,9,10,11]. A portion of the arachidonic acid (20:4n-6) released from the PAF precursor 1-*O*-alkyl-2-arachidonoyl-GPC by cPLA2α may be metabolized to prostaglandins and leukotrienes by the cyclooxygenase (COX) and lipoxygenase (LOX) pathways, respectively (Figure 6). Accumulation of arachidonic acid in 1-*O*-alkylacyl-GPC is favorable for the simultaneous synthesis of PAF and various eicosanoids in inflammatory cells. As stated earlier, supplementation of eosinophilic leukemia cells with DHA decreased the arachidonic acid content in 1-*O*-alkyl-2-acyl-GPC via competition with the CoA-independent transacylation reaction [77] and resulted in a reduction in PAF formation. These results suggest that phospholipid remodeling by CoA-independent transacylation is important for the synthesis of not only eicosanoids but also PAF in accelerated inflammation.

## 7. Possible Molecular Mechanisms of CoA-Independent Transacylation Reactions

Little is known about the molecular mechanisms of CoA-independent transacylation, such as the enzymes responsible for the reaction, which have yet to be purified; these enzymes are difficult to solubilize due to the high sensitivity of their enzymatic activity to various detergents [18]. Additionally, the genes encoding these enzymes have not yet been identified.

**Figure 7:**
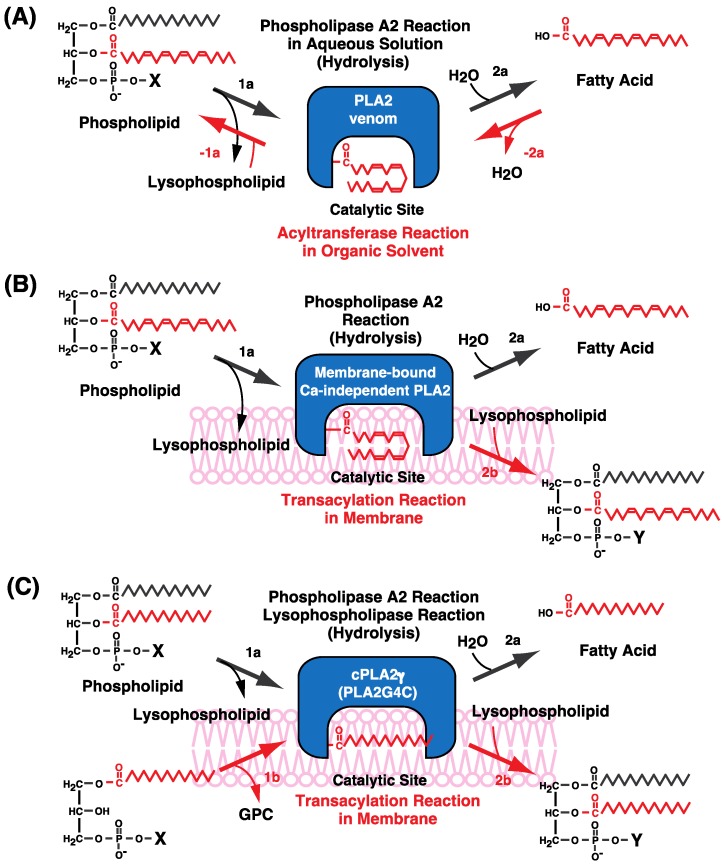
Possible mechanism of acyltransferase and transacylation activity catalyzed by PLA2. (**A**) Acyltransferase activity of venom PLA2 in a low-polarity organic solvent. We hypothesize that an intermediate fatty acyl–enzyme complex mediates the PLA2 reaction, which includes formation of a fatty acyl–enzyme complex by the half-reaction of PLA2 (step 1a) and fatty acid transfer from the complex to a water molecule (step 2a). Acyltransferase activity is demonstrated in the reverse PLA2 reaction and consists of steps −2a and −1a (red arrows). (**B**) Proposed model of the CoA-independent transacylation system. We hypothesize that a Ca^2+^-independent and membrane-bound PLA2 catalyzes the CoA-independent transacylation reaction, which consists of two steps: formation of a fatty acyl–enzyme complex as an intermediate by the half-reaction of PLA2 (step 1a) and transfer of a fatty acid from the acyl–enzyme complex to a lysophospholipid (step 2b), which is the reverse of step 1a. (**C**) Lysophospholipase/transacylation and CoA-independent transacylation reactions catalyzed by cPLA2γ. cPLA2γ can catalyze CoA-independent transacylation (described in panel B) as well as the lysophospholipase/transacylation reaction. Lysophospholipase/transacylation involves two steps: formation of a fatty acyl–enzyme complex by the half-reaction of lysophospholipase (or PLA1) (step 1b), and fatty acid transfer from the acyl–enzyme complex to lysophospholipid (step 2b). The transferred fatty acid is shown in red. X and Y represent parts of polar headgroups of phospholipids.

It was previously reported that several forms of extracellular secreted PLA2 (sPLA2), including pancreatic and venom PLA2s, operated in a reverse reaction in low-polarity solvents [78]—that is, free fatty acids were transferred to LPC (1-acyl-GPC) to form PC (Figure 7A, red arrows). Results indicated that PLA2 could catalyze the acyltransferase reaction under specific conditions. This prompted us to examine the potential contribution of PLA2 to acyltransferase and transacylation activities in membranes, which resemble the environments of low-polarity solvents that exclude water molecules, although a transacylation reaction was not reported in the earlier study [78].

We propose that CoA-independent transacylation reactions are catalyzed by enzymes belonging to the PLA2 family. Based on the characteristics of the CoA-independent transacylation system (Section 4 and Section 5), we speculate that Ca^2+^-independent and membrane-bound PLA2 proteins that selectively transfer C20–22 PUFAs including arachidonic acid are involved in this process (Figure 7).

The CoA-independent transacylation reaction comprises two steps: the first is the formation of the fatty acyl–PLA2 complex by the half-reaction of PLA2 (step 1a in Figure 7B), and the second is the transfer of fatty acids from this complex to lysophospholipids (step 2b in Figure 7B), which is the reverse reaction of step 1a. The fatty acyl–PLA2 complex can also react with a water molecule to complete the hydrolytic (PLA2) reaction (step 2a). It is critical that CoA-independent transacylation activity be present in the membrane fraction, including the microsomes, and nearly absent from the cytosolic fraction [18]. The catalytic site should be in a hydrophobic environment within the membrane to limit access to water molecules, thereby inhibiting hydrolysis and promoting transacylation.

It has been reported that CoA-independent transacylation involves enzyme(s) that are biochemically and pharmacologically distinct from cPLA2α and the 14-kDa sPLA2 [79]. This is consistent with our hypothesis that the enzyme in question is a membrane-bound and non-soluble PLA2 (Figure 7B).

## 8. Intracellular PLA2

We propose that CoA-independent transacylation is catalyzed by enzymes belonging to the PLA2 family—specifically, intracellular PLA2s. Before discussing the candidate enzymes, we first describe intracellular PLA2, which hydrolyzes glycerophospholipids at the *sn*-2 position to release fatty acids and lysophospholipids. As stated earlier (Figure 2), PLA2 is involved in the first step of the deacylation–reacylation cycle (Lands cycle) as well as in the synthesis of lipid mediators such as eicosanoids or PAF (Figure 6).

**Figure 8:**
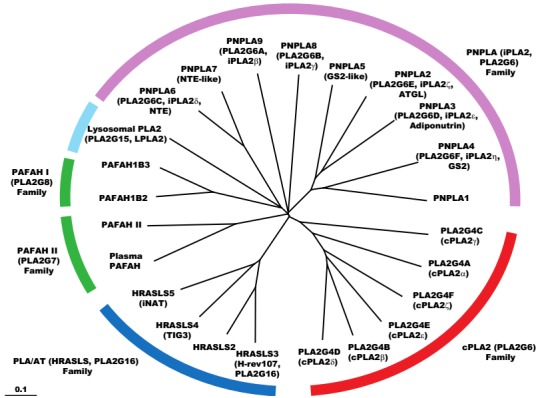
Phylogenetic tree of related phospholipases. Enzymes in the cPLA2, patatin-like PLA (iPLA2), PLA/AT, and intracellular and extracellular PAF-AH families are included in the tree. Amino acid sequences of each enzyme were obtained from GenBank and SwissProt and were aligned using the ClustalW program distributed by the DNA Data Bank of Japan. The phylogenetic tree was generated using the TreeView program. Branch lengths represent the evolutionary distances between sequence pairs.

The PLA2 enzyme superfamily consists of over 30 enzymes, with members such as sPLA2, cPLA2, Ca^2+^-independent PLA2 (iPLA2), PAF-AH, and lysosomal PLA2 classified according to localization (extracellular vs. intracellular), sequence homology, or biochemical characteristics. A systematic group numbering system has been proposed for these enzymes [80]. Figure 8 shows a phylogenetic tree of intracellular and related PLA2s.

**Figure 9:**
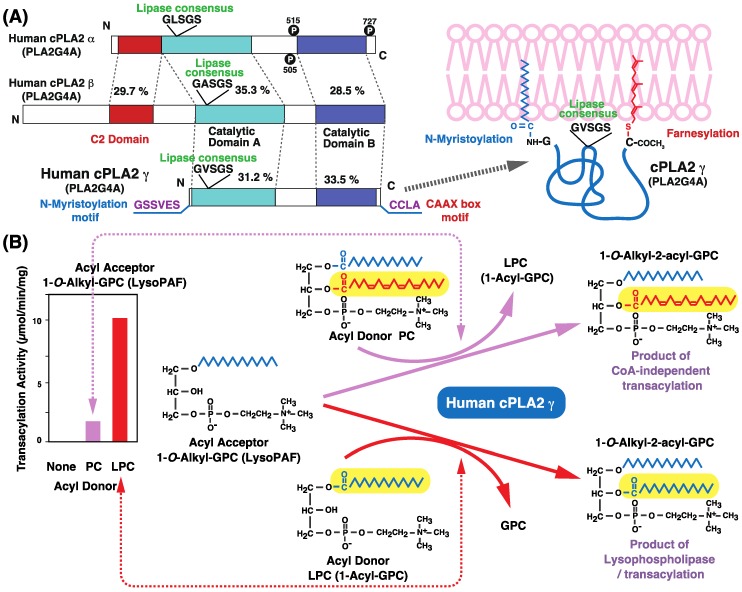
Structure and transacylation activity of cPLA2γ (PLA2G4C). (**A**) Schematic representation of sequence homologies among the primary types of cPLA2 (PLA2G), containing two homologous catalytic domains flanked by gene-specific sequences. A lipase consensus sequence (GXSGS) is located at the N-terminus of domain A. cPLA2α and cPLA2β both have an N-terminal C2 domain that is involved in Ca^2+^-dependent phospholipid binding. Human cPLA2γ has a CAAX box and putative N-myristoylation site at the C- and N-termini, respectively. cPLA2α can be phosphorylated at Ser505 by mitogen-activated protein kinases (MAPKs), at Ser515 by Ca^2+^/calmodulin-dependent protein kinase II, and at Ser727 by MAPK-interacting kinase 1 or a closely related isoform. Post-translational modifications such as C-terminal farnesylation and N-terminal N-myristoylation are depicted. (**B**) Transacylation activity of cPLA2γ. Purified cPLA2γC-FLAG was incubated with [^3^H]alkyl-GPC in the absence or presence of PC or LPC as acyl donors; the products were analyzed by thin-layer chromatography and the radioactivity of [^3^H]alkylacyl-GPC was measured. cPLA2γ exhibits CoA-independent transacylation activity for 1-*O*-alkyl-GPC and uses both phospholipids (upper, CoA-independent transacylation) and lysophospholipids (lower, lysophospholipase/transacylation) as the acyl donor, with preference for the latter. The transferred fatty acyl moiety is shown on a yellow background.

cPLA2α was identified as a key enzyme in eicosanoid synthesis [4,65,66,67,81,82,83] that selectively hydrolyzes arachidonic acid-containing phospholipids when cells are activated by extracellular stimuli, thereby stimulating eicosanoid synthesis. It is also involved in PAF synthesis (Figure 6). cPLA2α (PLA2G4A) harbors the conserved GXSXG sequence for lipase activity as well as a calcium-dependent lipid-binding domain that resembles the C2 domain of calcium-dependent protein kinase C enzymes (Figure 9). cPLA2α also has several serine residues that are phosphorylated by protein kinases, and it is activated by intracellular Ca^2+^ via C2 domain-mediated membrane binding and phosphorylation in response to extracellular stimuli. A study using transgenic mice lacking cPLA2α demonstrated the importance of prostaglandin and leukotriene synthesis in the allergic reaction and in parturition [84].

cPLA2 (PLA2G4) family enzymes were identified based on their sequence similarity to cPLA2α [85,86,87]. cPLA2β (PLA2G4B), cPLA2δ (PLA2G4D), cPLA2ε (PLA2G4E), and cPLA2ζ (PLA2G4F) were found to form the *cPLA2β* gene cluster, and all have both the lipase consensus sequence (GXSXG) and the C2 domain [87] (Figure 8).

Another enzyme belonging to the cPLA2 (PLA2G4) family, cPLA2γ (PLA2G4C), was identified as an ortholog of cPLA2α [85,86,88,89,90,91,92,93] and contains a lipase consensus sequence but lacks the C2 domain (Figure 9). Interestingly, cPLA2γ was found to contain a prenylation motif (CAAX box) at its C-terminus and a putative myristoylation motif at its N-terminus [85,86,88,89,90,91,92,93]. The subcellular localization of cPLA2γ also differs from those of other cPLA2 family members: it is detected in membrane fractions, including in the endoplasmic reticulum. In addition to having Ca^2+^-independent PLA2 activity, cPLA2γ exhibits PLA1 [85] and lysophospholipase [88,89,92,93] activities.

Another important group of intracellular PLA2s is the Ca^2+^-independent PLA2 family (iPLA2, PLA2G6) (Figure 8) [80,94,95,96,97,98], also known as the patatin-like phospholipase domain-containing protein (PNPLA) family [99]. Like cPLA2α, iPLA2β (PLA2G6A, PNPLA9) is also a serine-based enzyme, but its PLA2 activity was shown to be Ca^2+^ independent and was not specific to arachidonic acid-containing phospholipids [94,95,96,97]. iPLA2β is thought to play a pivotal role in phospholipid remodeling, including in the deacylation–reacylation cycle (Figure 2), since its inhibition by bromoenollactone reduces arachidonic acid incorporation into phospholipids in P388D1 macrophages [94], phorbol ester-differentiated U937 macrophage-like cells [95], and uterine stromal cells [96]. iPLA2-mediated generation of LPC (1-acyl-GPC) is associated with increased incorporation of arachidonic acid in PC (diacyl-GPC). In addition, iPLA2β has been implicated in lysophospholipid accumulation under hypoxic conditions; this can lead to the aggravation of arrhythmia, given the abundance of iPLA2β and its substrate, plasmalogen, in the myocardium [100,101,102].

Another isoform of iPLA2, iPLA2γ (PLA2G6B, PNPLA8), is a membrane-bound enzyme that may also be involved in the deacylation step of phospholipid remodeling. Recombinant iPLA2γ exhibits PLA1 and PLA2 activities depending on substrate type (Figure 8) [103]. Purified iPLA2γ was shown to hydrolyze oleic acid at the *sn*-2 position of 1-stearoyl-2-oleoyl-GPC, suggesting that it possesses PLA2 activity. Mass spectrometric analyses demonstrated that purified iPLA2γ readily hydrolyzed saturated or monounsaturated aliphatic groups at either the *sn*-1 or -2 positions of phospholipids. In addition, purified iPLA2γ liberated arachidonic acid from the *sn*-2 position of 1-*O*-alkenyl-GPC (plasmenylcholine). In contrast, incubation of iPLA2γ with 1-palmitoyl-2-arachidonoyl-GPC resulted in the rapid release of palmitic acid (hexadecanoic acid, 16:0) and selective accumulation of 2-arachidonoyl LPC (2-arachidonoyl-GPC), which was not further metabolized by iPLA2γ. These results indicate that iPLA2γ has PLA1 activity when arachidonic acid is present at the *sn*-2 position of diacyl PC.

Other iPLA2 (PLA2G6, PNPLA) family enzymes include lysophospholipases (PNPLA6 and PNPLA7) and triacylglycerol lipases (PNPLA1-5) (Figure 8) [80,98,99].

PAF-AHs are PLA2s that hydrolyze PAF and oxidized phospholipids (Figure 6 and Figure 8) [74,75,76,80,104]. PAF-AH I (PLA2G8) is an intracellular PAF-AH. The catalytic subunits PLA2G8A and PLA2G8B (also termed α1 and α2 subunits) form homo- and heterodimers. PAF-AH II (PLA2G7B) is another type of intracellular PAF-AH belonging to the plasma PAF-AH (PLA2G7A) family [74,75,76,80,105,106]. These enzymes hydrolyze not only PAF but also oxidized phospholipids—i.e., those that are oxidatively modified to contain short- or medium-chain fatty acids.

Lysosomal PLA2 (LPLA2, PLA2G15) is a PLA2 enzyme located in lysosomes that exhibits Ca^2+^ independence and has an acidic pH optimum [80,107]. It is also referred to as lysophospholipase 3 (LYPLA3), since it also exhibits lysophospholipase activity. The same enzyme has also been named lecithin-cholesterol acyltransferase (LCAT)-like lysophospholipase (LLPL) because this enzyme has 49% amino acid sequence identity to LCAT.

The PLA/AT (PLA2G16) family comprises a novel group that includes the Ca^2+^-independent N-acyltransferase (iNAT), which is involved in the synthesis of N-acyl PE [108]. These enzymes belong to the lecithin-retinol acyltransferase family and are negative regulators of the proto-oncogene Ras; indeed, iNAT is also known as HRAS-like suppressor (HRASLS) 5. Other family members include H-Rev107 (HRASLS3), HRASLS2, and tazarotene-induced gene (TIG) 3 (HRASLS4) [109,110,111]. These enzymes exhibit PLA1 or PLA2 activities to release free fatty acids from glycerophospholipids, with a preference for hydrolysis at the *sn*-1 position [109,110,111].

## 9. cPLA2γ Catalyzes CoA-Independent Transacylation Reactions that Transfer Arachidonic Acid to Ether-Linked Lysophospholipids

We previously suggested that cPLA2γ (PLA2G4C) may be responsible for CoA-independent transacylation [92,93]. cPLA2γ (PLA2G4C) was identified as an ortholog of cPLA2α (PLA2G4A) [85,86], whose activity was shown to be Ca^2+^ independent due to the absence of a C2 domain that is conserved in other cPLA2 (PLA2G4) family enzymes and is involved in Ca^2+^-dependent lipid binding. cPLA2γ is membrane bound owing to the presence of lipidation motifs, including a C-terminal CAAX farnesylation motif (Figure 9A,B). The membrane-bound and Ca^2+^-independent properties of cPLA2γ are similar to those of the CoA-independent transacylation system.

We examined whether cPLA2γ catalyzes CoA-independent transacylation using 1-*O*-[^3^H]alkyl-GPC (lysoPAF) as a substrate (Figure 9C) [92,93]. When purified cPLA2γ was incubated with 1-*O*-[^3^H]alkyl-GPC, 1-*O*-[^3^H]alkyl-GPC was acylated in the presence of PC (1-palmitoyl-2-arachidonyl-GPC). The transfer of arachidonic acid to ether-linked lysophospholipids was confirmed using 2-[^14^C]arachidonic acid-containing PC or PE as the acyl donor [93]. A schematic of the reaction is depicted in Figure 9C (right) and shows that cPLA2γ promoted CoA-independent transacylation of 1-*O*-alkyl-GPC (Figure 7C, reactions 1a + 2b). A farnesylation-defective mutant also exhibited comparable CoA-independent transacylation activity, suggesting that farnesylation is not essential for transacylation activity.

## 10. cPLA2γ Exhibits High Lysophospholipase/Transacylation Activity

Although cPLA2γ can catalyze CoA-dependent transacylation, it also exhibits lysophospholipase/transacylation activity (Figure 9). Lysophospholipase A hydrolyzes lysophospholipids to release fatty acids (Figure 7C, reactions 1b + 2a), whereas lysophospholipase/transacylation involves the transfer of cleaved fatty acids to lysophospholipids to form diradyl phospholipids (Figure 7C, reactions 1b + 2b).

When purified cPLA2γ was incubated with [^14^C]LPC (1-[^14^C]palmitoyl-GPC), [^14^C]palmitic acid was released, suggesting that cPLA2γ catalyzes the lysophospholipase reaction (Figure 7C, reactions 1b + 2a) [92,93]. In addition, [^14^C]PC was formed; analysis of the product [^14^C]PC by digestion with snake venom PLA2 revealed [^14^C]palmitic acid at both the *sn*-1 and -2 positions of [^14^C]PC. These results indicate that cPLA2γ catalyzed the transfer of [^14^C]palmitic acid at the *sn*-1 position of [^14^C]LPC to the hydroxy group at the *sn*-2 position of another [^14^C]LPC, confirming that [^14^C]LPC acts as both acyl donor and acceptor in the lysophospholipase/transacylation reaction (Figure 7C, reactions 1b + 2b) [92,93].

1-*O*-Alkyl-GPC acts only as an acyl acceptor due to the resistance of the alkyl ether bond to cleavage. The addition of LPC increased the acylation activity of 1-*O*-alkyl-GPC rather than that of PC (Figure 9B), suggesting that cPLA2γ prefers lysophospholipids (lysophospholipase/transacylation) to phospholipids (CoA-dependent transacylation reaction) as acyl donors (Figure 9B) [92,93]. CoA-independent transacylation and lysophospholipase/transacylation differ in terms of the acyl donor: the former uses phospholipids such as PC and PE, whereas the latter uses LPC. The lysophospholipase/transacylation activity of cPLA2γ was markedly higher than its CoA-independent transacylation activity. cPLA2γ also exhibited higher lysophospholipase activity (sequential reactions, steps 1b and 2a in Figure 7C) than PLA2 activity (sequential reactions, steps 1a and 2a in Figure 7C) [92,93], which may explain the ratio of each transacylation reaction (step 1a vs. 1b). These results indicate that cPLA2γ is not the enzyme responsible for CoA-independent transacylation-mediated accumulation of arachidonic acid in ether-linked phospholipids.

The physiological roles of cPLA2γ remain unclear. We also predict that cPLA2γ has important functions in the heart because it is highly expressed in this tissue [85,86]. In addition, lysophospholipids including lysoplasmalogen have been shown to accumulate in ischemic heart tissue [112,113,114]. Under hypoxic conditions, Ca^2+^-independent PLA2 (iPLA2β) is thought to be involved in lysophospholipid accumulation, since iPLA2β and its substrate plasmalogen are abundant in the myocardium [100,101,102]. Lysoplasmalogen as well as LPC are also known to trigger cardiac arrhythmia [112,113,114]. In iPLA2β transgenic mice, cardiac ischemia enhances iPLA2β activity, leading to increased lysophospholipid accumulation and aggravation of arrhythmia [102]. Given that the lysophospholipase and transacylation reactions of cPLA2γ can reduce the levels of toxic lysophospholipids, we hypothesize that cPLA2γ has a protective role against arrhythmia [92,93].

## 11. Other Enzymes that Catalyze Transacylation Reactions

Our group has investigated the enzymes responsible for CoA-independent transacylation [92,93]. Although cPLA2γ can catalyze this reaction, it also exhibits high lysophospholipase/transacylation activity (Figure 9B). Below, we discuss the possibility that other PLA2s participate in CoA-independent transacylation (Table 3).

cPLA2α (PLA2G4A), another cPLA2 family enzyme, was also shown to exhibit transacylation activity [115]. However, cPLA2α preferentially uses lysophospholipids as substrates in the transacylation reaction, transferring the fatty acids in LPC to another LPC to yield PC in the lysophospholipase/transacylation reaction (Table 3). Ca^2+^ is essential for cPLA2α-catalyzed PLA2 activity and for the binding of PLA2 to the membrane or micelles where the substrate is located. However, cPLA2α-catalyzed lysophospholipase activity towards micelles was not stimulated by Ca^2+^, and cPLA2α-catalyzed lysophospholipase/transacylase activity was low.

Another PLA2 isoform may participate in the CoA-independent transacylation system. The 30-kDa PLA2 belonging to the 14-3-3 protein family catalyzes the cleavage of the *sn*-2 arachidonic acid in phospholipids though the formation of a stable acyl–enzyme complex as an intermediate [116]. The occurrence of this arachidonic acid–enzyme complex may suggest that arachidonic acid in the complex to specifically transfer to putative nucleophilic acceptors including water (PLA2 activity) and lipids (transacylation activity).

**Figure 10:**
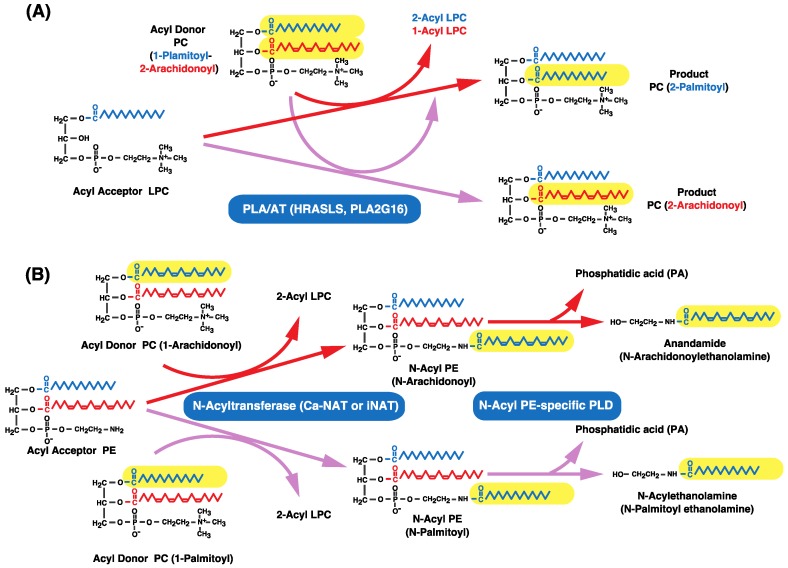
CoA-independent transacylation and biosynthesis of anandamide and related N-acylethanolamines by PLA/AT family enzymes. (**A**) PLA/AT family enzymes catalyze CoA-independent transacylation. Fatty acids at the *sn*-1 or -2 positions of acyl donor phospholipids are transferred to LPC (1-acyl-GPC), with preference for *sn*-1 over *sn*-2 fatty acids. (**B**) Fatty acids at the *sn*-1 positions of phospholipids are transferred to the amino group of PE to form N-acyl PE by NATs. N-acyl PEs are hydrolyzed by N-acyl PE-specific PLD to form N-acylethanolamines. Anandamide is an N-arachidonoyl species of N-acylethanolamine whose biosynthesis involves a rare fatty acid—i.e., arachidonic acid at the *sn*-1 position of a phospholipid. The transferred fatty acid is shown on a yellow background.

The PLA/AT family enzymes [HRASLS3 (H-rev107), HRASLS4 (TIG3), and HRASLS2] were also shown to exhibit CoA-independent transacylation activity [108,109,110,111]. The fatty acids at the *sn*-1 or *sn*-2 positions of donor phospholipids are transferred to LPC in a Ca^2+^-independent manner, with preference for the transfer of *sn*-1 rather than *sn*-2 fatty acids. These enzymes also exhibit PLA1 or PLA2 activities, releasing free fatty acids from glycerophospholipids with a preference for hydrolysis at the *sn*-1 position [108,109,110,111]. Although these enzymes could catalyze CoA-independent transacylation, they are not thought to be involved in the acylation of ether-linked lysophospholipids with arachidonic acid based on their positional preference (Figure 10A) (Table 3).

The PLA/AT family enzymes are reported to exhibit another type of transacylation activity for the synthesis of N-acyl PE (Ca^2+^-independent N-acyltransferase, iNAT) (Table 3) [108]. iNAT can catalyze the transfer of fatty acids at the *sn*-1 position of PC to an amino group of PE in the absence of Ca^2+^ to yield N-acyl PE (Figure 10B). H-Rev107 (HRASLS3), HRASLS2, and TIG3 (HRASLS4) also exhibit such transacylation activity [109,110,111]; HRASLS2 showed high iNAT activity, while the activities of H-rev107 and TIG3 were low. Recently, cPLA2ε (PLA2G4E) was also shown to catalyze a similar but Ca^2+^-dependent transacylation reaction [117]. This Ca^2+^-dependent NAT activity (Ca-NAT) was consistent with the presence of the conserved C2 domain involved in Ca^2+^-dependent lipid binding in cPLA2ε. Ca-NAT and iNAT activities are thought to involve N-acylethanolamines such as anandamide (Figure 10B) [7,8,108,109,110,111,118,119,120,121].

Type 2 intracellular PAF-AH (PAF-AH II) may exhibit transacylation activity (Figure 8) (Table 3) [122,123,124,125]. The transfer of the acetyl group from PAF to lysophospholipid acceptors was demonstrated in the HL-60 membrane fraction (CoA-independent transacetylation) [122,123]. Purification and cDNA cloning revealed that the transacetylation activity was due to PAF-AH II [124,125]. PAF-AH II catalyzed transacetylation from PAF to 1-radyl-GPC, 1-radyl-GPE, 1-acyl-GPS, 1-acyl-GPI, 1-radyl-GP, and lysoplasmalogen. Sphingosine also served as an acceptor. PAF-AH-catalyzed transacetylation showed broader acceptor specificity than the CoA-independent transacylation system, which transfers long-chain acyl moieties. These differences in substrate donor and acceptor specificities suggest that the CoA-independent transacetylase and CoA-independent transacylation systems are composed of two different enzymes.

Plasma-type PAF-AH was also shown to exhibit transacylation activity (Figure 8) (Table 3) [126]. The occurrence of transacetylase activity was demonstrated in human plasma low-density lipoproteins, which transfer acetyl groups from PAF to lysophospholipids. This enzyme was able to transfer not only the acetyl moiety but also short-chain acyl moieties including propionyl (C3:0), butanoyl (C4:0), and valeroyl (C5:0) residues.

Lysosomal PLA2 (LPLA2, PLA2G15) was also reported to catalyze transacylation reactions (Figure 8) (Table 3). Abe et al. identified this enzyme as having novel activity for the synthesis of 1-*O*-acyl-ceramide [127]. Activity was characterized by transfer of the *sn*-2 fatty acid of PC to the 1-hydroxyl group of N-acetylsphingosine. The product of this reaction was 1-*O*-acylceramide (1-*O*-acyl-N-acetylsphingosine). Further characterization employing the recombinant protein revealed that the enzyme predominantly exhibited PLA2 activity rather than ACS activity. Thus, this enzyme is now known as lysosomal PLA2 (LPLA2) [128,129].

## 12. Concluding Remarks and Future Directions

In this review, we summarized the characteristics and physiological significance of the CoA-independent transacylation system, which is involved in fatty acid remodeling of glycerophospholipids and in the accumulation and enrichment of arachidonic acid in alkyl ether-linked phospholipids in the brain, heart, and various inflammatory cells. This system is also important for the metabolism of eicosanoids and PAF. Despite its importance, the component enzyme(s) have not yet been identified. We described here the reaction mechanism of CoA-independent transacylation and discussed the involvement of Ca^2+^-independent and membrane-bound PLA2. In Section 11, we discussed how many lipases, including PLA2s, can catalyze transacylation, even although their substrate specificities differ. This lends support to the rationale behind our proposed model (Figure 7) for the involvement of PLA2 in CoA-independent transacylation.

A detailed analysis of enzymological data revealed that cPLA2γ is not the enzyme responsible for CoA-independent transacylation, since it prefers to use lysophospholipids rather than phospholipids as acyl donors. However, this in vitro substrate specificity may be due to the solubilities of lyso and diradyl phospholipids as acyl donors. Further detailed characteristics of cPLA2γ will be needed, including analyses using cPLA2γ-knockdown or -knockout macrophages or neural cells, to investigate the involvement of cPLA2γ in the accumulation of arachidonic acid in ether-linked phospholipids.

Identifying the enzyme(s) involved in the CoA-dependent transacylation system will require a combination of highly specific probes—including substrates/inhibitors with covalent modifications such as photoaffinity labeling—and sensitive methodologies such as liquid chromatography–tandem mass spectrometry. This information will improve our understanding of the physiological significance of alkyl ether phospholipids and the biosynthesis of PAF and eicosanoids.

## Figures and Tables

**Table 1:** Classification and abbreviations of glycerophospholipids and lysophospholipids discussed in this review.

Class	Subclass	Chemical	Other Names
**Diradyl glycerophospholipid**			
Choline glycero-phospholipid	1, 2-Diacyl	1, 2-Diacyl-*sn*-glycero-3-phosphocholine (1,2-Diacyl-GPC)	Phosphatidylcholine (PC)
	1-*O*-Alkyl-2-acyl	1-*O*-Alkyl-2-acyl-*sn*-glycero-3-phosphocholine (1-Alkyl-2-acyl-GPC)	Plasmanylcholine
			Alkyl phosphatidylcholine
	1-*O*-Alkenyl-2-acyl	1-*O*-Alkenyl-2-acyl-*sn*-glycero-3-phosphocholine (1-Alkenyl-2-acyl-GPC)	Plasmenylcholine
			Alkenyl phosphatidylcholine
			Choline plasmalogen
Ethanolamine glycero-phospholipid	1, 2-Diacyl	1, 2-Diacyl-*sn*-glycero-3-phosphoethanolamine (1,2-Diacyl-GPE)	Phosphatidylethanolamine (PE)
	1-*O*-Alkyl-2-acyl	1-*O*-Alkyl-2-acyl-*sn*-glycero-3-phospho-ethanolamine (1-Alkyl-2-acyl-GPE)	Plasmanylethanolamine
			Alkyl phosphatidylethanolamine
	1-*O*-Alkenyl-2-acyl	1-*O*-Alkenyl-2-acyl-*sn*-glycero-3-phospho-ethanolamine (1-Alkenyl-2-acyl-GPE)	Plasmenylethanolamine
			Alkenyl phosphatidyethanolamine
			Ethanolamine plasmalogen
Serine glycero- phospholipid	1, 2-Diacyl	1, 2-Diacyl-*sn*-glycero-3-phosphoserine (1,2-Diacyl-GPS)	Phosphatidylserine (PS)
Inositol glycerol-phospholipid	1, 2-Diacyl	1, 2-Diacyl-*sn*-glycero-3-phosphoinositol (1,2-Diacyl-GPI)	Phosphatidylinositol (PI)
**Radyl lysophospholipid**			
Choline glycerol-phospholipid	1-Acyl	1-Acyl-*sn*-glycero-3-phosphocholine (1-Acyl-GPC)	Lysophosphatidylcholine (LPC)
			1-Acyl lysoPAF
	1-*O*-Alkyl	1-*O*-Alkyl-*sn*-glycero-3-phosphocholine (1-Alkyl-GPC)	1-Alkyl lysophosphatidylcholine (1-Alkyl LPC)
			Lyso platelet activating factor (LysoPAF)
	1-*O*-Alkenyl	1-*O*-Alkenyl-*sn*-glycero-3-phosphocholine (1-Alkenyl-GPC)	1-Alkenyl lysophosphatidylcholine (1-Alkenyl LPC)
			Choline lysoplasmalogen
Ethanolamine glycerol-phospholipid	1-Acyl	1-Acyl-*sn*-glycero-3-phosphoethanolamine (1-Acyl-GPE)	1-Acyl lysophosphatidylethanolamine (1-Acyl LPE)
	1-*O*-Alkyl	1-*O*-Alkyl-*sn*-glycero-3-phosphoethanolamine (1-Alkyl-GPE)	1-Alkyl lysophosphatidylethanolamine (1-Alkyl LPE)
	1-*O*-Alkenyl	1-*O*-Alkenyl-*sn*-glycero-3-phosphoethanolamine (1-Alkenyl-GPE)	1-Alkenyl lysophosphatidylethanolamine (1-Alkenyl LPE)
			Ethanolamine lysoplasmalogen
Serine glycerol-phospholipid	1-Acyl	1-Acyl-*sn*-glycero-3-phosphoserine (1-Acyl-GPS)	1-Acyl lysophosphatidylserine (1-Acyl LPS)
	2-Acyl	2-Acyl-*sn*-glycero-3-phosphoserine (2-Acyl-GPS)	2-Acyl lysophosphatidylserine (2-Acyl LPS)
Inositol glycerol-phospholipid	1-Acyl	1-Acyl-*sn*-glycero-3-phosphoinositol (1-Acyl-GPI)	1-Acyl lysophosphatidylinositol (1-Acyl LPI)
	2-Acyl	2-Acyl-sn-glycero-3-phosphoinositol (2-Acyl-GPI)	2-Acyl lysophosphatidylinositol (2-Acyl LPI)

**Table 2:** Acyltransferases and transacylation systems involved in fatty acid remodeling.

Acyltransferases and Transacylation Systems	Cofactor	Acyl Donor	Acyl Acceptor	Fatty Acid Transferred	Enzyme(s) Involved
Acyl-CoA:Lysophospholipid Acyltransferase		Acyl-CoA	LPC, LPE, LPS, LPI		AGPAT family
					MBOAT family
CoA-Independent Transacylation System	None	Phospholipids	1-*O*-alkyl-GPC	C20, C22 PUFA at *sn*-2	Not Identified
		PC, PE	1-*O*-Alkenyl-GPE		
CoA-Dependent Transacylation System	CoA	Phospholipids	LPC, LPE, LPS, LPI	20:4, 18:2 at *sn*-2 18:0 at *sn*-1	Involvement of Acyl-CoA Acyltransferases
		PI > PC, PE			
Lysophospholipase/Transacylation	None	LPC, LPE	LPC, LPE		cPLA2γ (PLA2 G4C)

**Table 3:** Phospholipases that catalyze transacylation reactions.

Family	Candidates	Cofactor	Reactions	Acyl Donor	Acyl Acceptor	Features
cPLA2 (PLA2G4)	cPLA2γ (PLA2 G4C)	None	CoA-independent transacylation	Phospholipids PC, PE	1-*O*-alkyl-GPC1-*O*-Alkenyl-GPE	Low activity?
			Lysophospholipase/transacylation	LPC, LPE	LPC, LPE 1-*O*-alkyl-GPC1-*O*-Alkenyl-GPE	Clearance of lysophospholipid?
	cPLA2α (PLA2 G4A)	None	Lysophospholipase/transacylation	Phospholipids PC, PE	LPC, LPE	
	cPLA2ε (PLA2G4E)	Ca^2+^	N-acyltransferase	PC, PE	PE	Anandamide synthesis
iPLA2 (PNPLA, PLA2G6)	iPLA2ε (PNPLA3)	None	Triacylglycerol lipase/transacylase	Triacylglycerol	Acylglycerol	Triacylglycerol degaradation/synthesis
	iPLA2ζ (PNPLA2)					
	iPLA2η (PNPLA4)					
PLA/AT (HRASLS, PLA2G16)	HRASLS5 (iNAT)	None	N-acyltransferase	PC, PE	PE	Anandamide synthesis
	HRASLS3 (H-Rev107)					
	HRASLS2		CoA-independent transacylation	PC, PE	LPC, LPE	Preference to *sn*-1 fatty acid than *sn*-2 ones as acyl donor
	HRASLS4 (TIG3)					
14-3-3 protein family	30 kDa PLA2	None	Transacylation reaction?	Phospholipids PC, PE		
PAFAH II (PLA2G7)	PAF-AH II	None	Transacetylation	PAF, Oxidized Phospholipid	1-*O*-Alkenyl-GPE, sphigosine	Transfer of short chain fatty acid
	Plasma PAF-AH	None	Transacetylation	LPC, LPE	LPC, LPE	Transfer of short chain fatty acid
Lysosomal PLA2	LPLA2 (PLA2G15)	None	Transacylation	PC	1-*O*-Alkenyl-GPE, ceramide	
